# Decompression strain in parachute jumpmasters during simulated high-altitude missions: a special reference to preoxygenation strategies

**DOI:** 10.1007/s00421-023-05173-9

**Published:** 2023-03-23

**Authors:** Ola Eiken, Antonis Elia, Frode Gottschalk, Mikael Gennser, Rickard Ånell

**Affiliations:** Division of Environmental Physiology, Swedish Aerospace Physiology Centre, KTH Royal Institute of Technology, Stockholm, Sweden

**Keywords:** Altitude decompression sickness, Altitude preoxygenation, Decompression bubble precursor, Decompression bubble formation, High-altitude parachuting, Hypobaric preoxygenation

## Abstract

**Purpose:**

Military parachute operations are often executed at high altitude, from an unpressurized aircraft compartment. Parachute jumpmasters (JM) are thus regularly exposed to 29,500 ft for 60 min. The aim was to investigate the decompression strain during a simulated JM mission at high altitude and to compare two strategies of preoxygenation, conducted either at sea-level or below 10,000 ft, during ascent to mission altitude.

**Methods:**

Ten JM completed, on separate occasions, a 45-min preoxygenation either at sea-level (normobaric: N) or 8200ft (hypobaric: H), followed by exposure to 28,000 ft for 60 min, whilst laying supine and breathing 100% oxygen. At min 45 of the exposure to 28,000 ft, the JM performed 10 weighted squats. Decompression strain was determined from ultrasound assessment of venous gas emboli (VGE) during supine rest (5-min intervals), after three unloaded knee-bends (15-min intervals) and immediately following the weighted squats. The VGE were scored using a six-graded scale (0–5).

**Results:**

In condition H, two JM experienced decompression sickness (DCS), whereas no DCS incidents were reported in condition N. The prevalence of VGE was higher in the H than the N condition, at rest [median(range), 3(0–4) vs 0(0–3); *p* = 0.017], after unloaded knee-bends [3(0–4) vs 0(0–3); *p* = 0.014] and after the 10 weighted squats [3(0–4) vs 0(0–3); *p* = 0.014]. VGE were detected earlier in the H (28 ± 20 min, *p* = 0.018) than the N condition (50 ± 19 min).

**Conclusions:**

A preoxygenation/altitude procedure commonly used by JM, with a 60-min exposure to 28,000 ft after pre-oxygenation for 45 min at 8200 ft is associated with high risk of DCS. The decompression strain can be reduced by preoxygenating at sea level.

## Introduction

Parachuting is occasionally undertaken from very high altitudes [high altitude high opening; (HAHO) or high altitude low opening (HALO) parachuting]. For tactical reasons, such high-altitude missions are particularly common in military settings, with drops of troops and/or equipment being conducted from altitudes exceeding 32,000 ft. For obvious reasons, the aircraft compartment from which the drops are executed is unpressurized, and consequently, personnel engaged in HAHO/HALO operations may be at risk of developing altitude decompression sickness (ADCS) (Ottestad et al. 2017).

Judging by anecdotal information, symptoms compatible with type I DCS (pain in muscles or joints) have been noted on several HAHO/HALO missions in the Swedish Airforce (SwAF), and it appears that predominantly the parachute jumpmasters (JM) have been affected. Presumably, a higher ADCS incidence among the JMs than the parachuters, is associated with the fact that the JMs commonly are exposed to high altitude for extended time periods and typically perform strenuous muscle exercise in conjunction with the drops; it is well established that both the exposure duration and performance of physical exercise during the exposure, increase the risk of ADCS (Barcroft et al. 1931; Conkin et al. 2013; Jauchem 1988; Stepanek and Webb 2008). SwAF parachuters regularly undertake missions at 29,500 ft, during which the JM may be exposed to the drop altitude for 60 min. A preliminary in-flight study (Ånell et al., unpublished results) confirmed that such exposure was associated with considerable decompression strain, with 3 of 4 JMs exhibiting high scores of venous gas emboli (VGE) at the drop altitude.

The risk of ADCS can be reduced, not merely by limiting the altitude and exposure duration (Ernsting 1995), but also by breathing 100% oxygen (O_2_) prior to the high-altitude exposure (preoxygenation), which depletes the body nitrogen (N_2_) depots and thereby reduces the formation of inert gas bubbles upon decompression (for refs, see Stepanek and Webb 2008). Since O_2_ is a metabolic gas, decompression bubbles predominated by O_2_ are inherently unstable, and hence, preoxygenation substantially reduces the risk of ADCS (for refs see Pilmanis et al 2005; Stepanek and Webb 2008). The standard procedure is to preoxygenate at sea-level atmospheric pressure (Stepanek and Webb 2008), but currently it is common that, to save time, aircrew, including JM and HAHO/HALO parachuters, preoxygenate during ascent to the drop altitude. It is not well examined if, and in what manner, such preoxygenation at low-to-moderate altitude (≤ 8200 ft) will affect the decompression strain during the subsequent high-altitude exposure.

Accordingly, the aims of the present study were to, in a hypobaric chamber, determine (i) the decompression strain during simulation of a typical HAHO/HALO mission, comprising a 60-min exposure to 28,000 ft, and (ii) whether the decompression strain is affected by the altitude at which the 45-min preoxygenation procedure is conducted.

## Methods

### Subjects

Ten healthy men, all serving as JM in the SwAF, volunteered to participate. Their mean (± SD) age, height and mass were 43 ± 6 years, 1.84 ± 0.06 m and 89 ± 6 kg, respectively. All JMs were briefed in detail about the study purpose, the experimental procedures and the potential risks and benefits, prior to giving their written consent. Each JM was aware that he was entitled to terminate any trial or to withdraw from the study at any time without providing any reason. Ethics approval for the study was granted by the regional Human Ethics Committee in Stockholm, Sweden (approval no. 2020-001606), and all experimental procedures conformed to the Declaration of Helsinki.

### Equipment and procedures

The experiments were undertaken in a 21-m^3^ hypobaric chamber (AB Motala Verkstad, Sverige) at the Division of Environmental Physiology, KTH, Royal Institute of Technology, Stockholm, Sweden. All experiments were monitored and recorded using a video/audio surveillance system (JVC MI 5000 Victor Company, Japan). Each JM and the inside experimenter, were breathing O_2_ via pressure demand-regulated full facemasks (Atmosphere, Poseidon Diving Systems AB, Sweden), connected to 50-L compressed gas bottles (filling pressure: 200 ATM) via pressure-reduction valves. To avoid high levels of O_2_ in the chamber, the JM’s expired gas was collected in a confined space of the chamber, via a respiratory hose (diameter 38 mm) connected to the facemask; the space was ventilated regularly, and the O_2_ content in the chamber’s main compartment was monitored continuously (Datex Normocap 200 Oxy, Datex, Finland) throughout each experiment.

The occurrence of VGE was detected in the right cardiac ventricle from four-chamber cardiac ultrasound images, using a phased-array transducer (1–5 MHz) and an ultrasound system (CX50, Philips Ultrasound Bothell, USA). Prevalence of VGE was evaluated from the cardiac ultrasound images using a six-point scale (0 = no visible bubbles, 1 = occasional bubbles, 2 = at least one bubble every fourth heartbeat, 3 = at least one bubble every heartbeat, 4 = at least one bubble/cm^2^, 5 = “whiteout”, single bubbles cannot be discriminated) (Eftedal et al. 2007; Nishi et al. 2003). All cardiac images/videos were stored on a computer hard drive (Latitude E5530, Dell, USA), and subsequently analysed by the inside experimenter as well as another experienced sonographer as described in detail elsewhere (Elia et al. 2022). Symptoms of DCS and/or other pain/discomfort were graded every five min, using a 10-point ratio scale (Borg 1982).

Cardiac output (CO) and heart rate (HR) were measured continuously using an impedance system (PhysioFlow PF07 system, Enduro, Manatec Biomedical, France), and data were stored on a computer (Latitude E5530, Dell, USA). Capillary oxyhaemoglobin saturation was monitored continuously, both in the JM and the inside experimenter, using pulse oximeters (Radical 7 Monitor MASIMO SET, USA), with the sensor placed on a finger; the signals were merely used for surveillance and were hence not stored or further analysed.

### Experimental protocol

**Fig. 1:**
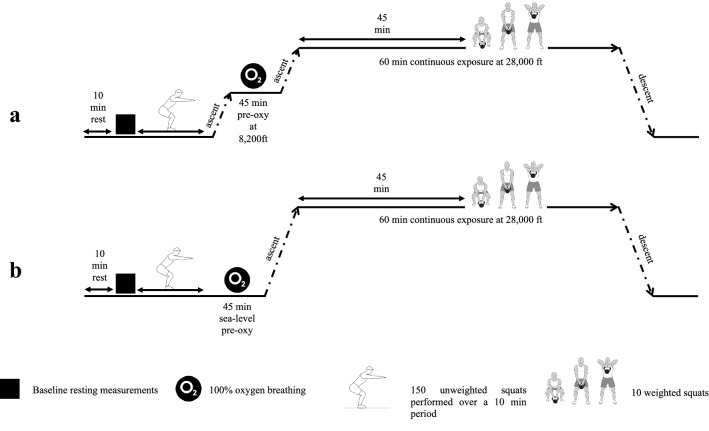
Schematic illustration of the experimental protocol, with unweighted squats followed by a 45-min preoxygenation at 8200 ft (**a**; hypobaric condition; H) or at sea level (**b**; normobaric condition; N), and subsequently by a 60-min exposure to a simulated altitude of 28,000 ft, during which the JM was laying horizontally except for at minute 45 when he stood up and performed 10 weighted squats

The experimental protocol is schematically depicted in Fig. 1. Each JM was investigated on two occasions, separated by 4–7 days. On each occasion, the JM inspired pure O_2_ during a 45-min preoxygenation period as well as during the 60-min simulated altitude exposure [chamber pressure corresponding to an altitude of 28,000 ft asl (8534 m)] and during the ascent and descent periods. On one occasion, the preoxygenation was performed at sea-level atmospheric pressure [Normobaric (N) condition] and on the other occasion, preoxygenation was performed at a chamber pressure corresponding to 8200 ft asl [2500 m; Hypobaric (H) condition]. The order of the two trials was alternated among the JMs in a counter-balanced fashion. For the individual JM, the two trials were performed at approximately the same time of the day and the ultrasound examinations were performed by the same inside experimenter accompanying the JM throughout each chamber exposure (Elia et al. 2022).

The JMs were instructed to avoid strenuous exercise (48 h) and nicotine intake (4 h) before each experiment. Immediately prior to an experiment, the JM, who was dressed in trousers and sneakers, performed 150 unweighted squats over a 10-min period, in an attempt to equalise the amount of existing gaseous micronuclei in the circulation (Dervay et al. 2002). Thereafter, he was instrumented with six pre-gelled electrodes on the thorax and neck, for impedance- and electro-cardiography recordings. The JM then donned the facemask and commenced the idle preoxygenation period. In the H condition, chamber pressure was reduced to the simulated altitude of 8200 ft, which was maintained throughout the preoxygenation period. After 45 min of preoxygenation, chamber pressure was reduced and the 60-min exposure to a simulated altitude of 28,000 ft ensued. The ascent and descent rates (including during ascent to 8200 ft in the H condition) were 5000 ft/min. During the initial 45 min at 28,000 ft, the JM was laying horizontal, either supine or on his left side. At min 45, he stood up and performed 10 weighted deep squats lifting a 20-kg “kettle bell” with both arms extended straight out (≈ 90° angle) from the trunk. The procedure, which was executed in ≈ 1 min, was to simulate the work performed by the JM in conjunction with a HAHO/HALO drop. After the kettle-bell exercise, the JM reassumed the horizontal position.

During the exposure to 28,000 ft, CO and HR values were recorded continuously, whereas the prevalence of VGE was determined intermittently, at 5-min intervals. In conjunction with every third VGE measurement (i.e. every 15 min), the JM performed three unloaded knee bends while in the left decubitus position, to provoke release of bubbles adhering to vascular endothelium into the venous circulation (Gennser et al. 2012; Jankowski et al. 2004). Every 5 min, the JM was also asked about any symptoms of DCS (pain or discomfort), and if any, to rate their severity using the ratio scale. DCS was not the primary endpoint of the altitude exposure, which nonetheless was a priori set to be terminated if a JM reported DCS symptoms or the inside experimenter graded a persistent (two consecutive measurements) VGE score ≥ 4.

The inside experimenter completed a 60-min preoxygenation period at sea level prior to the start of the experimental trials and resumed preoxygenating for a further 45-min before the ascent to 28,000 ft, either at sea level (N condition) or at 8200 ft (H condition).

### Analyses

The Kisman integrated severity score (KISS) was calculated according to the following formula:

$$\mathrm{KISS}= \left[100/{(4}^{\alpha }\left({t}_{n}-{t}_{1}\right))\right]\cdot \left[\left({t}_{2}-{t}_{1}\right)\left({d}_{2}^{\alpha }+{d}_{1}^{\alpha }\right)+\dots +\left({t}_{n}-{t}_{n-1}\right)\left({d}_{n}^{\alpha }+{d}_{n-1}^{\alpha }\right)\right]/2,$$

where *t*_*i*_ = time of observation in min after reaching altitude (for time points 1 to *n*), *d*_*i*_ = ultrasound score (grades 0 to 5) observed at time *t*_*i*_, and *α* = 3 (the parameter α takes into account that the bubble grade is not a linear measure of bubble quantity) (Kisman et al. 1978; Nishi et al. 2003; Pontier and Lambrechts 2014).

All data were statistically analysed using IBM SPSS Statistics software version 28 (IBM Corp., Armonk, NY, USA). Wilcoxon signed-rank tests were used to assess inter-condition differences in VGE scores. In addition, the VGE scores were converted (time integrated) using the KISS equation, and differences within (supine rest vs. knee-bend provocations) and between conditions were assessed using Wilcoxon signed-rank tests.

Paired sample *t* tests were used to compare the two conditions regarding time taken for the first VGE to be detected in the right ventricle. Repeated measures ANOVA with post hoc Tukey’s contrast comparisons were used to assess for differences between resting baseline measurements and other collection time points for heart rate and cardiac output. Unless otherwise stated, data are reported as means ± SD and significance was accepted at *p* < 0.05, and *p* = 0.000 was reported as *p* < 0.001. GraphPad Prism version 7.0c (GraphPad Software Inc., La Jolla, CA, USA) was used to construct figures.

## Results

During the simulated exposure to 28,000 ft, VGE were observed in 9 out of 10 JM in the H condition, whereas in the N condition, VGE were only detected in 4 out of 10 JM. Moreover, in the H condition, 2 out of 10 JM developed DCS (95% CI 4.5–64.5%), with local pain occurring after 40 min at 28,000 ft [right knee (grade 4/10] for one of the JM, and after 55 min [left ankle (grade 4/10] for the other JM; both these JM exhibited a VGE score of 4 in conjunction with the onset of pain. The high-altitude exposure was then terminated, and the pain disappeared during descent, at 21,000 and 17,000 ft for the knee and ankle case, respectively. By contrast, in the N condition, all experimental sessions were completed, and none of the simulated altitude exposures resulted in DCS symptoms (95% CI 0–41%).

### Peak venous gas-emboli scores

**Fig. 2:**
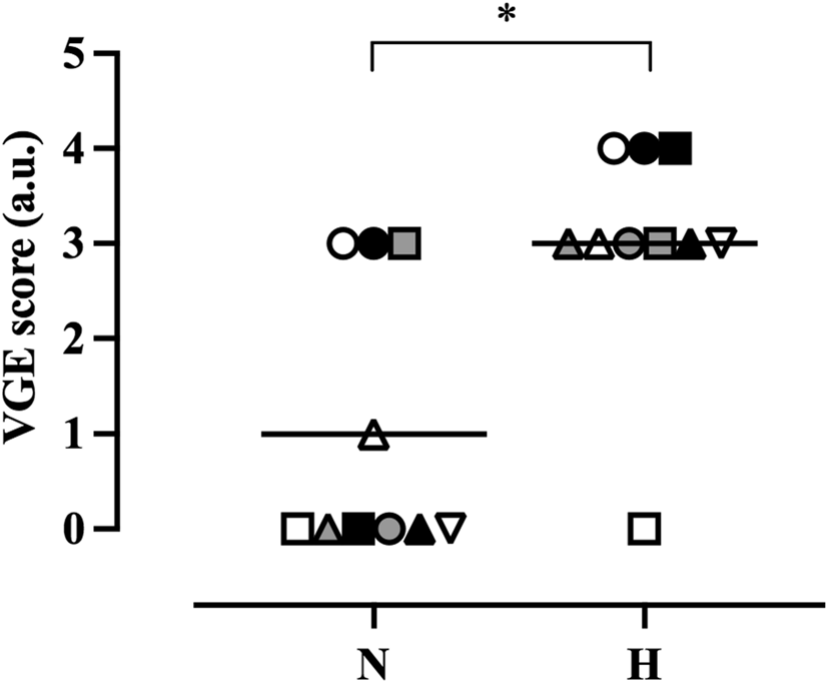
Peak VGE scores in the normobaric (N) and hypobaric (H) conditions during the 60-min continues exposure to 28,000 ft. *Denotes significant difference (*p* < 0.05) between conditions, symbols are individual values, and black lines represent median values. Wilcoxon signed-rank test; *n* = 10

Peak VGE scores were higher in the H than N condition, both during horizontal rest and after horizontal knee-bend provocations (*p* = 0.017, *p* = 0.014, respectively). Specifically, at rest, median VGE score was 3 (range 0–4) and 0 (range 0–3) in the H and N conditions, respectively; after the knee-bend provocations, median VGE score was 3 (range 0–4) in the H and 0 (range 0–3) in the N condition (Fig. 2).

VGE scores were increased (*p* = 0.039) by the altitude exercise intervention in the H condition (pre, 3 [0–3]; post, 3 [0–4]) (Table 1). Contrastingly, no differences (*p* = 0.157) in VGE scores could be discerned before vs after the exercise intervention in the N condition (pre, 0 [0–3]; post, 0 [0–3]) (Table 1).

**Table 1:** Individual peak VGE scores before and after the altitude exercise intervention

JM	Pre-exercise		Post-exercise	
	N condition	H condition	N condition	H condition
1	3	3	3	4
2	1	3	3	4
3	0	0	0	0
4	0	0	0	3
5	0	3	0	3
6	0	2	0	3
7	0	3	0	3
8	1	1	3	3
9	0	3	0	3
10	0	4	0	−
Median	0	3	0	3

In the H condition, JM 10 developed DCS prior to the altitude exercise intervention, and hence is not included in the post-exercise column

Abbreviations: *N* normobaric condition, *H* hypobaric condition

VGE scores were higher in the H than the N condition, both prior to [H condition, 3 (0–3); N condition, 0 (0–3); *p* = 0.038] and following the upright exercise intervention [H condition, 3 (0–4); N condition, 0 (0–3); *p* = 0.014] (Table 1). Additionally, time before the first VGE was detected at 28,000 ft, was shorter (*p* = 0.018) in the H (28 ± 20 min) than the N condition (50 ± 19 min).

### Integrated venous gas-emboli scores

**Fig. 3:**
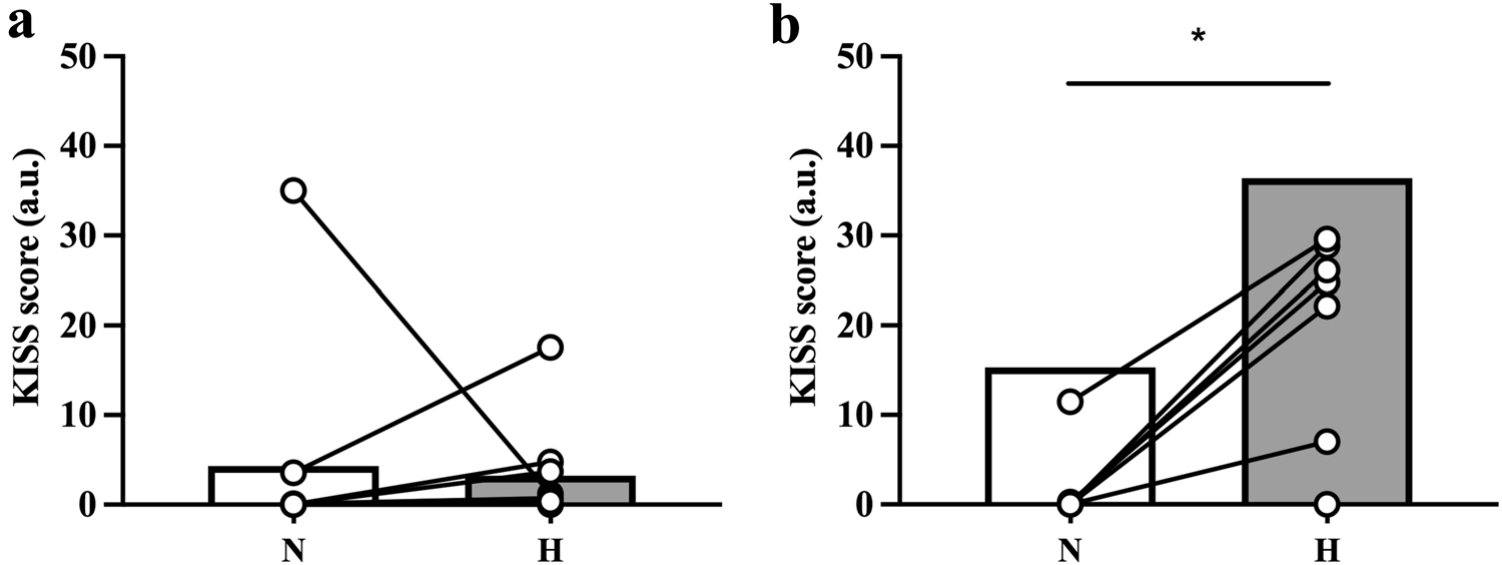
Individual (white dots) and mean KISS scores (bar charts) before (**a**) and after the 10 weighted squats (**b**) in the normobaric (N) and hypobaric (H) condition. Wilcoxon signed-rank test; *n* = 9

KISS were higher after knee bends than at rest in the H condition (*p* = 0.025) but not in the N condition (*p* = 0.273). In addition, after the knee-bend provocations, KISS was higher (*p* = 0.051) in the H (mean ± SD: 20 ± 13 a.u.) than the N condition (7 ± 13 a.u.), whereas, during supine rest there was no inter-condition difference (N condition, 5 ± 12 a.u.; H condition, 7 ± 10 a.u., *p* = 0.161) (Fig. 3). Likewise, following the exercise intervention at altitude, KISS were higher in the H (36 ± 35 a.u., *p* = 0.012) than in the N condition (15 + 28 a.u.), whereas, no inter-condition differences were discerned prior to the exercise modality (N condition, 4 ± 12 a.u.; H condition, 3 ± 6 a.u., *p* = 0.237).

### Cardiovascular variables

There were no significant differences between conditions in any of the cardiovascular variables neither at rest [HR, 71 ± 9 (N), 69 ± 8 (H), p = 0.327; CO, 6.3 ± 0.7 (N), 5.6 ± 1 (H), *p* = 0.341], nor during the knee-bend provocations [HR, 81 ± 8 (N), 78 ± 9 (H), p = 0.667; CO, 6.9 ± 1.1 (N), 6.6 ± 1 (H), *p* = 0.389] nor during the altitude exercise modality [HR, 112 ± 14 (N), 104 ± 6 (H), *p* = 0.619; CO, 12 ± 2.7 (N), 11 ± 2.4 (H), *p* = 0.292].

## Discussion

Present results demonstrated that a 60-min exposure to 28,000 ft after a 45-min hypobaric preoxygenation was associated with a substantial risk of developing DCS, with two of the ten subjects exhibiting DCS symptoms during the high-altitude exposure. In addition, VGE occurred earlier and the VGE scores were higher in the H condition, with several subjects approaching the end-point threshold for decompression strain, i.e. exceeding three on the six-graded scale (Fig. 2). In the N condition, by contrast, none of the subjects developed DCS, and the VGE scores were in general low, even though certain individuals exhibited high VGE density, particularly after the weighted deep squats (Table 1).

A central question is why the decompression strain upon acute exposure to altitude appears to be greater when preceded by a hypobaric instead of a normobaric preoxygenation. The pathophysiology of DCS is not fully understood. For instance, despite that it is well documented that the formation of decompression bubbles exhibits large inter-individual (Cialoni et al. 2015) and significant intra-individual (Ånell et al. 2022) variability, the mechanisms underlying such variabilities are but partially known. It is, however, generally accepted that altitude-induced decompression bubbles develop from precursors in terms of existing gaseous micronuclei (Doolette 2009; Vann et al. 1980; Yount and Strauss 1976), since de novo formation of decompression bubbles in a homogenous liquid requires a pronounced supersaturation of gas molecules, corresponding to a pressure drop of more than 100 ATM (Doolette 2009). It is likewise generally agreed that preoxygenation protects against DCS by facilitating diffusion between the body tissues and the alveolar gas compartment, thereby reducing the partial pressure of N_2_ and increasing the partial pressure of O_2_ in the tissues (Pilmanis et al. 2005; Stepanek and Webb 2008; Webb et al. 1993, 1998). Any decompression-dependent gas bubbles formed during the following altitude exposure will then comprise a greater share of O_2_ molecules and a lesser share of N_2_ molecules than if the exposure was not preceded by a preoxygenation procedure. Therefore, and because decompression bubbles predominated by O_2_ are inherently less stable than those predominated by N_2_ (Ånell et al. 2021b; Blogg et al. 2003; Hyldegaard and Madsen 1997), preoxygenation reduces the bubble prevalence during altitude exposure (Ånell et al. 2021b; Hyldegaard and Madsen 2007). In addition, preoxygenation may reduce the incidence of ADCS by diminishing bubble precursors/nuclei and hence the Boyle-expansion formation of bubbles during the following high-altitude exposure (Arieli et al. 2011; Blatteau et al. 2006; Bosco et al. 2010; Tikusis and Gerth 2003). Also, the finding that whole-body vibration substantially reduces VGE formation upon a succeeding altitude exposure, supports the notion that reducing the number of micronuclei is an efficient means of preventing altitude-induced decompression bubbles (Elia et al. 2021).

It must be assumed that in the present investigation, the diffusion gradient for N_2_ from the body tissues to the pulmonary gas compartment was similar during the O_2_ breathing in the two experimental conditions, whereas the blood transport capacity for physically dissolved N_2_ was presumably somewhat lower during the hypobaric than the normobaric preoxygenation (Van Liew et al. 1993). Thus, in the pressure range where preoxygenation is normally carried out, the oxygen window (i.e., the difference between the ambient pressure and the sum of the gas partial pressures in the venous blood) is directly proportional to the inspired oxygen partial pressure (P_I_O_2_) (Van Liew et al. 1993). However, even during the hypobaric preoxygenation, the P_I_O_2_ was of the same magnitude as the initial N_2_ pressure in the tissues, which means that the JMs were within the extended oxygen window. This concept, the extended oxygen window (P_eow_ = P_I_O_2_), has been employed to carry out safe decompressions from N_2_/O_2_ and air saturation dives (Kot et al. 2015). It appears unlikely, therefore, that any inter-condition difference in N_2_ transport capacity was of sufficient magnitude to significantly affect the whole-body wash-out rate for N_2_ (Van Liew et al. 1993).

Nevertheless, the decompression strain during the following exposure to 28,000 ft was markedly greater in the H than in the N condition. A contributing factor was without doubt the rapid initial decompression from 1 to 0.75 ATM, inducing a 33% Boyle-dictated expansion of any micronuclei. Even though the subjects were breathing 100% O_2_ during the 1.5-min ascent to 8200 ft and onwards, it must be assumed that this initial bubble expansion was dominated by inward diffusion of N_2_ molecules, rendering the bubbles more stable. Thus, in the H condition, ascent commenced from a gas-saturation condition with stable N_2_-dominated micronuclei in the tissues. The resulting N_2_ supersaturation upon decompression would tend to fill the micronuclei with further N_2_. Another mechanism rendering the hypobaric preoxygenation less efficient would be the reduced surface tension of the expanded bubbles in accordance with the Young–Laplace equation, which would act to stabilise the decompression bubbles. Notably, without a period of oxygenation before a rapid ascent, it appears that, even during O_2_ breathing, the growth of decompression VGE are to large extent attributable to N_2_ diffusion into bubbles from tissue compartments with half-times for N_2_ washout exceeding 90 min (Conkin et al. 1987; Lundin 1960).

We have found no accounts in the literature of previous studies on the effects of hypobaric vs normobaric preoxygenation. However, a situation analogue to the present hypobaric preoxygenation is decompression following saturation dives. It appears that decompression from dives with long bottom times, where saturation dives are a prime example, are safer when using deep stops (Gutvik et al. 2008). It has been hypothesised that the DCS-protective mechanism of the early stops is a maintained high pressure in the micronuclei or decompression bubbles, favouring an outward gas diffusion. Hence, decompressions from long dives should target optimisation of inert gas transport from bubbles, rather than whole-body washout of dissolved inert gas (Gutvik et al. 2008). Altitude decompressions are in essence decompression from a saturation situation. Notably, it appears that also during prolonged exposure to high altitude whilst breathing O_2_, an efficient means to reduce the long-term decompression strain is to facilitate the gas exchange in the initial decompression bubbles from N_2_ to O_2_ predominated, by a short early excursion to moderate altitude (Ånell et al. 2021a).

From a practical viewpoint, present results clearly show that normobaric preoxygenation is preferable to hypobaric ditto, to reduce the decompression strain during high-altitude missions, conducted either without pressurisation of the aircraft cabin (e.g. parachute operations) or with reduced cabin pressure (e.g. fighter aircraft operations). Notwithstanding, it is our impression that in-flight preoxygenation during ascent has become common practice in several air forces, including the SwAF.

### Study delimitations

Even though decompression-induced VGE are sensitive markers of decompression strain, symptoms of ADCS are typically not caused by venous bubbles but rather by bubbles formed in muscles, tendons, joints or, on rare occasions, in the central nervous system (Stepanek and Webb 2008). Thus, a considerably larger study cohort is needed to establish to what extent the risk of ADCS is increased by preoxygenating at 8200 ft rather than at sea level. Preferably, such study should be confirmed by in-flight measurements. Since it is well established that preoxygenation substantially reduces the risk of ADCS (Stepanek and Webb 2008), we reasoned that it would have been unethical to compare the present two conditions with a control condition without any preoxygenation. The present experiments were performed in men only, because there are no female JMs in the SwAF. It is likely that the qualitative study outcome—that high-altitude decompression strain is greater after hypobaric than normobaric preoxygenation—would be the same in female test subjects (Webb et al. 2003), but this remains to be investigated.

## Conclusions

In conclusion, the present study showed that a preoxygenation/altitude procedure commonly employed by Swedish military jumpmasters, with a 60-min exposure to 28,000 ft after preoxygenation for 45 min at 8200 ft, is associated with high risk of DCS. It appears that the high-altitude decompression strain can be reduced by conducting the preoxygenation at sea level. Presumably, the increased decompression strain after hypobaric preoxygenation, reflects a stabilisation of existing gaseous micronuclei by Boyle expansion induced by the initial ascent to 8200 ft.

## Data Availability

Data supporting the study findings may be requested from the corresponding author (O.E.), but are not publicly available since they contain information that could compromise the privacy of the research participants.

## Abbreviations

ADCS: Altitude decompression sickness
DCS: Decompression sickness
H: Hypobaric
HAHO: High-altitude high opening
HALO: High-altitude low opening
JM: Jumpmaster
KISS: Kisman integrated severity score
N: Normobaric
P_I_O_2_: Inspired partial pressure of oxygen
SwAF: Swedish Airforce
VGE: Venous gas emboli
